# Burnout among disability activists in England: Insights from the Oldenburg Burnout Inventory

**DOI:** 10.1371/journal.pone.0342846

**Published:** 2026-02-25

**Authors:** Lyla Adwan-Kamara, Fabrício M. Fialho, Amanpreet Ahluwalia, Husnara Zaman, Michelle Daley, Peter Beresford OBE

**Affiliations:** 1 Atlantic Fellow for Social and Economic Equity, London School of Economics and Political Science, London, United Kingdom; 2 Department of Sociology, HSE University, Moscow, Russia; 3 International Inequalities Institute, London School of Economics and Political Science, London, United Kingdom; 4 Birthing Ourselves, London, United Kingdom; 5 Lived experience expert,; 6 Disability Advice Service Lambeth, London, United Kingdom; 7 The Alliance for Inclusive Education (ALLFIE), London, United Kingdom; 8 School of Health Care, University of East Anglia (visiting professor), Norwich, United Kingdom; 9 Co-chair, Shaping Our Lives, London, United Kingdom; George Mason University, UNITED STATES OF AMERICA

## Abstract

**Objectives:**

To contribute to the understanding of burnout among Disabled activists in the disability movement - ‘disability activists’ - in England using the Oldenburg Burnout Inventory, and provide insights into the prevalence and features of burnout among this group.

**Methods:**

The self-completed online survey collected data on burnout among adults who self-identify as Disabled and as activists in the disability movement. The survey used questions on activism, socio-demographics, the Oldenburg Burnout Inventory for burnout assessment, and other attitudinal questions. The sample included 134 respondents recruited through disability-related organisations and disability-related activist networks. An important aspect of the method was oversight by a steering group of lived experience disability experts, and to prioritise survey accessibility.

**Results:**

A varied spectrum of impairment types was selected by respondents with the majority of respondents reporting long-term health conditions, and 74% reported having more than one impairment. Eighty-eight per cent of respondents reported that they undertake unpaid activism and 64% were undertaking paid activism. The Oldenburg Burnout Inventory has two dimensions – disengagement and exhaustion – which are strongly correlated (*ϕ* = 0.83), however, respondents differ significantly in their burnout levels in each dimension and tend to be significantly more exhausted than disengaged. Bivariate analysis shows that impairment type and lived experiences impact on the experience of burnout in ways that most of the other socio-demographic categories do not, respondents with energy-limiting conditions were particularly burnt out. Age and place of activism also make a difference in the experience of burnout, with younger disability activists and activists in charities not run by Disabled People having higher levels of burnout. Respondents self-assessed their level of burnout and results were closely correlated with the Oldenburg Burnout Inventory, suggesting that respondents understand whether they are burnt out and the intensity of their burnout. They also identified the key drivers of burnout which wasn’t their activism but rather the barriers that they face when confronted with ableism and the impact that ableist systems of oppression have on them.

**Conclusions:**

The survey findings raise a number of important points adding to our understanding of burnout among disability activists. Some respondents mentioned support from and solidarity with others as a protective factor from burnout, which should be further explored.

## Introduction

Since the late 1980s in the UK, across both Conservative, Labour, and coalition governments, Disabled People have been targeted and scapegoated in the pursuit of neoliberal ideology, government spending cuts and as part of the shift towards privatisation of welfare support [1,2]. This hostile environment has led to hardship and deaths of Disabled People, as well as being a driving force behind the founding of numerous campaigning organisations such as Black Triangle, Disabled People Against Cuts (DPAC) and many others [1].

This focus on addressing and trying to end the harms caused to Disabled People by government changes to welfare sits within the broader history of the vibrant Disabled People’s Movement in the UK since the 1960s, including the growth of organisations with a focus on independent living and anti-discrimination, led by and for Disabled People, termed Disabled People’s Organisations (DPOs), User Led Organisations (ULOs), and Centres for Independent Living (CILs) from the 1970s onwards [3].

Within this context, the social model of disability was developed, which states that it is society’s attitudes, infrastructure, transport and other disabling barriers which disable people, rather than their individual impairments. The social model was a tool used to think differently about the barriers in Disabled people’s lives and how to address them [4].

Despite some policy gains over the years, such as the introduction of the Disability Discrimination Act (later merged into the Equality Act 2010), organisations in the Disabled People’s Movement have seen significant funding losses in recent years, leading in some cases to closure of organisations as they compete with larger charities which are typically not run by Disabled People [5], and the loss of grants and contracts from local government as austerity took hold. Therefore, as demand for support by Disabled People who were losing access to life-saving support rose due to the re-design of a hostile benefits system and squeezed local governments, DPOs and CILs were simultaneously being defunded and closed down.

Despite the strongly political and activist nature of large parts of the Disabled People’s Movement in the UK [6], much of the literature focuses on the how and why of disability activism, without addressing the possible impact of activism, positive or negative, on activists’ wellbeing. For example, the Routledge Handbook of Disability Activism [7], does not cover the issue of burnout. Although work on the burnout of social justice activists exists, only limited literature addresses burnout among disability activists, with much of the existing work focussing on burnout from self-advocacy, for example in securing personal care or living independently [8], rather than a more political understanding of user-led disability activism.

A recent scoping review [8] identified that the literature has hardly engaged with the burnout of Disabled People, most frequently positioning them as the cause of burnout in others, such as caregivers [9]. An emerging body of evidence is exploring autistic burnout and suggests that this may be a separate type of burnout [10]. However, much of this literature focuses on the role and impact of masking in driving burnout, rather than considering autistic people as activists or the role their experience of activism may play in burnout.

Although the literature on burnout among disability activists is very limited, we can look to adjacent fields such as other types of activist burnout, or burnout of Disabled People in other contexts. For example, a US study of medical students with disabilities (their terminology) compared to medical students without disabilities identified higher levels of burnout in the former [11]. Several other recent studies among Disabled health professionals [12], carers [13], students [14], teachers [15] and athletes [16] conclude that they experience higher levels of burnout than non-disabled groups but very few of these studies take a social model approach, and most seem to consider disability itself driving higher burnout rather than contextual factors such as the barriers that Disabled people face, or the lack of adjustments made for them. At most, studies relate higher burnout to lack of support or disability understanding [12,14,15], with only a study on disabled caregivers giving an explicitly socio-political analysis with its clear rejection of stigmatizing narratives of disability as a burden [13].

There is a growing body of literature focussed on burnout among activists which provide some interesting reflections to help deepen our understanding of disability activism. Themes of secondary trauma when helping others address difficult experiences [17] or racial battle fatigue due to the cumulative effect of multiple oppressions [18] particularly resonate. Studies which explore burnout among activists with personal connections to their cause, such as anti-carceral [19] or racial justice activists [20], also provide a framework for understanding the experience of disability activists who may feel compelled to carry on despite feelings of burnout, or who may not as easily disconnect from the reality of their cause, as they live the consequences of that oppression daily.

Curious gaps remain in the literature, such as new studies on burnout among the disability sector workforce which don’t look at whether any of those workers are Disabled People [21,22]. In another example, an in-depth study on the experiences of youth activists gathered data on disability status which amounted to nearly 10% of study respondents, but chose not to analyse it [23].So, while we can draw parallels with the broader literature, many gaps remain given geographical and sectoral differences. Personal experience among most of the authors as part of the Disabled People’s Movement in the UK indicates that burnout among disability activists is widespread, but the lack of evidence inhibits addressing the issue. Therefore, the objective of this study is to contribute to the understanding of burnout among disability activists in England by employing the Oldenburg Burnout Inventory (OLBI) and providing insights into the prevalence and features of burnout among this group. This objective was largely achieved through the survey. The issues will be further explored in an upcoming paper on the qualitative component of the broader project which will provide an in-depth account of disability activists’ experiences of burnout and its causes.

## Methods

### Data collection

The study collected novel data on burnout among disability activists in England using an online survey. The self-completed online survey contained questions on activism, socio-demographics, the Oldenburg Burnout Inventory for burnout assessment, and other attitudinal questions.

There were three eligibility criteria to participate in the study: (i) Being an adult aged 18 and over (ii) who identified as being a Disabled Person, and (iii) being a disability activist in England. The survey introduction defined a disability activist as a Disabled Person doing campaigning, policy work, research, advice work, advocacy support or other types of activism. The survey also defined a Disabled Person as any person with an impairment from across the full spectrum of disability including people with mental health conditions or chronic conditions. Respondents were able to specify their understanding of their activism work and their impairments in the survey, see Table 1.

**Table 1:** Socio-demographic features of respondents.

Category	N	%
*Impairment type*		
Autistic spectrum	34	25.4
Deaf/hearing impairment	24	17.9
Energy-limiting condition	57	42.5
Mental health service user/survivor	66	49.3
Learning difficulty	13	9.7
Long-term health conditions	70	52.2
Neurodiversity	48	35.8
Physical impairment	68	50.7
Visual impairment/sight loss	12	8.9
Other	15	11.2
*Number of impairments*		
One	34	25.4
Two	25	18.7
Three	32	23.9
Four	17	12.7
Five	15	11.2
Six or more	11	8.2
*Type of activism*		
Paid activism	86	64.2
Unpaid activism	118	88.1
Unpaid activism alongside paid work	73	54.5
*Type of organisation (paid or unpaid)*		
Campaigning organisation	77	57.5
Centre for Independent Living	22	16.4
Disability Charity not run by Disabled People	22	16.4
User-led charity	82	61.2
Education / arts	15	11.2
Business and consultancy	19	14.2
Other	72	53.7
*Gender*		
Female	77	57.5
Male	40	29.8
Non-binary	12	9.0
Other / prefer not to say	5	3.7
*Age*		
18-24	2	1.49
25-34	40	29.9
35-44	26	19.4
45-54	33	24.6
55-64	21	15.7
65 and over	11	8.2
Prefer not to say	1	0.7
*Sexuality*		
Bi-sexual	21	15.7
Gay man	9	6.7
Gay woman / lesbian	6	4.5
Heterosexual	72	53.7
Other / prefer not to say	25	18.6
*Ethnicity*		
Asian or Asian-British	13	9.7
Black or Black-British	7	5.2
White British	77	57.5
Other White	21	15.7
Other ethnic group	13	9.7
Prefer not to say	3	2.2
*Religion*		
Christian	23	17.1
Muslim	8	6.0
No religion	76	56.7
Other / prefer not to say	28	20.9
*Class*		
Working class	46	34.3
Lower middle class	27	20.1
Middle class	40	29.9
Upper middle class	6	4.5
Prefer not to say / Other class or description	15	11.2

Fieldwork took place from 22 March to 10 June 2024. Respondents were recruited via convenience sampling initially, with some additional snowball sampling. To reach as many disability activists as possible, invitations to participate in the survey were disseminated through disability-related organisations such as DPOs and other disability-related activist networks. All organisations listed in both Disability Rights UK and Inclusion London’s lists of DPOs were contacted via email or via the contact form on their websites; the contact message included an explanation of the project’s objectives, a link to the online survey, and a request to disseminate it among staff and members. The project’s steering group also promoted the survey link among their networks.

Convenience sampling was used for practical utility as the overall population of disability activists in England is not known, and the study had a short timeframe for completion [24]. Using snowball sampling to support sampling is increasingly common when attempting to hear from marginalised or less reached groups who may be more difficult to identify systematically, or who face practical and other barriers to responding [25,26].

Dissemination was initially supported through social media (LinkedIn and X -– formerly Twitter); however, sharing the link on social media was discouraged following attacks by automated response accounts and ‘bots’.

A total of 945 completed responses were received, however, a large number of automated responses or ‘bots’ were excluded from the survey during data cleaning, resulting in a final sample of 134 respondents.

The cleaning protocol identified a range of factors which in combination marked a response as likely to be a bot when 3 or more factors were present. These included a flag by Qualtrics software, when the survey completion was less than 10 minutes, when the survey was completed between midnight and 6am. Flagged responses were then checked manually for nonsensical email addresses, incongruent responses, similarity to responses immediately before and after, use of language and contradictions between open and multiple-choice responses. These measures were developed in line with recent literature on identifying and managing bot attacks [27–30].

Robust approaches to data-validation can be considered especially important when working with under-represented groups, like disability activists, where the inclusion of fraudulent survey responses could result in misunderstanding of the situation and lead to faulty recommendations for action [30].

Some respondents appeared not to be ‘bots’ but were also excluded from the results based on their location such as Scotland, or USA. Others were excluded if they said they did neither paid nor unpaid activist work. Pre-testing responses were excluded.

### Selection of the instrument for burnout assessment

A range of instruments for the measurement of burnout have been advanced in the existing literature. One of the most widely used burnout scales is the Maslach Burnout Inventory (MBI) [31]. MBI and its variants have been extensively evaluated and used in burnout research (see, among many others, [32–34]). Its use was nevertheless discounted for this study primarily for its proprietary status, which prevented us from examining the survey questions in advance for suitability for this study’s purposes. In addition, the commercial nature of MBI is unappealing in the context of constrained research budgets, and it was unclear without further exploration whether the resulting data and tools would be fully shareable. The MBI has also been criticized for limitations in its framing and conceptualisation of burnout [35–37].

The Oldenburg Burnout Inventory (OLBI) and the Copenhagen Burnout Inventory (CBI) are two alternative instruments to assess burnout. CBI was not selected as a portion of the survey focuses on the respondent’s feelings towards their clients, which falls out-of-scope for this project [38]. Due to the broad definition of ‘activism’ considered in this study, it could not be determined whether respondents would find the client-related part of the scale relevant. For example, disability activists may be working in an advice or advocacy setting with clients that they support, or they may be part of a voluntary campaign group, such as Disabled People Against Cuts and taking part in protests or working on national policy issues.

The World Health Organisation has added burnout to its latest International Classification of Diseases (ICD-11) [39] and emphasises that it relates only to occupational settings. However, multiple studies exist validating OLBI across a range of settings, including unpaid groups [36,40,41] and different countries [36,42], suggesting good usability for the purposes of this study.

The OLBI features two scales, exhaustion and disengagement. The 16 questions have balanced positive (pro-trait) and negative (con-trait) wording, and the exhaustion scale is designed to assess both the cognitive and physical components of exhaustion [43]. The sixteen items are listed on Table 2.

**Table 2:** The Oldenburg Burnout Inventory items per subscale.

OLBI Dimension	Item	Item valence
Disengagement	D1. I always find new and interesting aspects in my work	Con-trait
Disengagement	D2. It happens more and more often that I talk about my work in a negative way	Pro-trait
Disengagement	D3. Lately, I tend to think less at work and do my job almost mechanically	Pro-trait
Disengagement	D4. I find my work to be a positive challenge	Con-trait
Disengagement	D5. Over time, one can become disconnected from this type of work	Pro-trait
Disengagement	D6. Sometimes I feel sickened by my work tasks	Pro-trait
Disengagement	D7. This is the only type of work that I can imagine myself doing	Con-trait
Disengagement	D8. I feel more and more engaged in my work	Con-trait
Exhaustion	E1. There are days when I feel tired before I arrive at work	Pro-trait
Exhaustion	E2. After work, I tend to need more time than in the past in order to relax and feel better	Pro-trait
Exhaustion	E3. I can tolerate the pressure of my work very well	Con-trait
Exhaustion	E4. During my work, I often feel emotionally drained	Pro-trait
Exhaustion	E5. After working, I have enough energy for my leisure activities	Con-trait
Exhaustion	E6. After my work, I usually feel worn out and weary	Pro-trait
Exhaustion	E7. Usually, I can manage the amount of my work well	Con-trait
Exhaustion	E8. When I work, I usually feel energised	Con-trait

### Making data collection accessible

Data was gathered in March-June 2024 through an online survey via Qualtrics. The design of the data collection instrument incorporated features to ensure that as many and as wide a variety of disability activists as possible would have the opportunity to complete the survey.

At the start of the study, a steering group of lived experience experts in disability, and who are involved in the Disabled People’s Movement, was established. The steering group focussed on review and refinement of the overall approach, avoiding harm, and reviewing overall method and outputs. The group advised on the implementation of the social model of disability approach, such as the language used, accessibility and pre-testing of the survey.

The Qualtrics interface was formatted to ensure minimum font size of 12 and increased size of arrows and instructions to support ease of use. To ensure maximum accessibility, a Word version of the questionnaire was available for respondents to download in case the Qualtrics version did not meet their accessibility needs.

An EasyRead version of the survey tool was also made available. The EasyRead version used accessible language and images to support comprehension. It was developed by an external supplier based on the original survey tool and reviewed by the steering group prior to use.

All respondents submitted their responses via the online tool, although they also had the option of submitting by email using the Word or EasyRead versions. Although the accessibility versions were not used to submit responses in the way anticipated, a broad mix of activists with different impairments did complete the survey, including people with learning difficulties or visual impairments, see Table 1. In addition, Two groups were talked through the survey prior to completion. This included one Deaf club where a sign language interpreter and Deaf member of the steering group used the EasyRead tool to explain the purpose of the survey and to clarify the concepts used. For example, ‘activism’ needed to be defined for the group because in British Sign Language it is visually similar to the sign for ‘activities’. Group members then completed the online survey independently. A leadership group at Inclusion London were also talked through the survey prior to completing it independently.

Prior to accessing the questionnaire, respondents were provided with a short summary of the context and objectives of the study. They were asked to consent to the survey and reminded they could withdraw from participation at any point. Respondents had to tick a consent box before proceeding with the survey. Additionally, respondents were asked to confirm they met the key eligibility criteria -- being a Disabled person who is a disability activist in England -- and then asked to provide demographic information. These respondents are referred to as disability activists throughout this paper.

Following eligibility, respondents completed the 16 OLBI questions. Following the OLBI, respondents were asked to identify the extent to which they felt they were burnt out on a 4-point scale, and to identify what they thought might be driving burnout through a multiple-choice list and open question. These additional questions to the OLBI were included based on research which recommends that surveys for assessing burnout include open questions as “people often put a lot of thought and effort into their comments, and the results can give valuable insights, especially if themes emerge across a wide range of responses” [44].

The online survey took on average 20 minutes to complete. This is longer than expected in the original survey design which anticipated a 10-minute response time. Two Deaf respondents took 3.5 hours and over 4 hours, respectively, to complete the survey. They appear to have kept the survey open during the Deaf club session where the survey was explained. Excluding these two responses, the average response time is nearly 17 minutes. The open responses to the survey were detailed and lengthy compared to other surveys the authors have been involved in, which may be driving the longer than expected response times.

Respondents who completed the survey were offered an e-voucher of £5. This was a ‘thank you’ payment to recognise people’s time and contribution to the project, based on the principle that people should be fairly compensated for their labour [45]. However, due to concerns that payments could affect respondents’ welfare benefits entitlement, all were offered the opportunity to have their thank you paid to a community group on their behalf instead. Of the 134 validated responses, 63 chose to donate their compensation. Respondents who requested the voucher provided their email addresses, which were later removed from the records during data analysis.

### Ethics

This study, including its data protection plan, received ethical approval from the London School of Economics’ Research Ethics Committee (reference number 323047).

### Statistical analysis

Prior to analysis, the collected data was cleaned to remove entries generated by bot attacks, resulting in a survey data set containing the abovementioned 134 respondents.

Due to the relatively small sample size, additional care has been taken in the reporting of uncertainty and in the assessment of statistical significance. Accompanying group means in the disengagement and exhaustion dimensions, we report standard errors (s.e.), 95% confidence intervals (95% CI), and their p-values based on 1,000 resamplings. In group comparisons, the mean differences (represented by the greek letter delta, Δ) are also reported with their respective measures of uncertainty, now calculated using permutation tests; we opted to assess the statistical significance of group mean differences using permutation tests (based on 1,000 permutations) because the Bonferroni correction can lead to severe penalties in small samples.

All the statistical analyses reported below were conducted using the open-source freeware software R 4.5 [46]. All R scripts used in the analysis are available upon request.

## Limitations

All of the possible instruments reviewed, including OLBI which was selected as most appropriate for this study, are self-reporting instruments. Although self-reporting can sometimes be considered a limitation, this approach was considered most appropriate for studies asking people about their personal experiences [47], especially in the context of the experiences of Disabled People who may experience being researched on about rather than with [48]. A second scale could have been included for comparison of results, however, this was also discounted due to the length of the survey. To off-set these limitations, in addition to the OLBI tool, the survey includes a single question to identify the extent to which respondents feel burnt out on a 4-point scale, and several open questions, which align closely to the OLBI findings.

Burnout research and tools have typically focused on work-related conditions and contexts, and this is the case with the Oldenburg Burnout Inventory. This project focuses on the diverse experiences of disability activists, some of whom may be unpaid, volunteering, or might not see their activism as work. Existing literature nevertheless indicates that OLBI can be used to assess burnout among volunteers [36,40,41]. The information and consent form that preceded the survey acknowledged this issue and prompted respondents to answer the questions specifically in relation to their activism. However, it cannot be ruled out that the participants also considered other factors whilst responding to the survey..

Some disability activists may be digitally excluded [49,50] and out of reach of surveys disseminated digitally through emails, newsletters and other networks and therefore not represented in the data, despite the attempts to reach as many disability activists as possible. In particular, sharing of the survey invitation by contacted organisations may have been uneven, and people not working or volunteering in the contacted organisations had fewer opportunities to become aware of the survey and decide whether or not to respond. The overall sampling approach is also limited by the fact that respondents are self-selecting and may have more motivation to reply, whether because they are burned out and want to share the experience or because they are not burned out and feel more able to reply. These factors limit the generalisability of the findings.

## Results and discussion

### Sample demographics

Table 1 outlines the socio-demographic features of respondents. The survey provided a drop down list and asked “Please select as many impairments or conditions which apply to your experience of disability. If I haven’t used the correct terminology, you can use your own language if you prefer”. The terms were not defined and some may overlap, therefore people could select as many options as they felt was suitable for them. A varied spectrum of impairment types was selected by respondents with the majority of respondents reporting long-term health conditions, followed by mental health conditions and physical impairments. Seventy-four per cent of respondents reported having more than one impairment.

Eighty-eight per cent of respondents reported that they undertake unpaid activism and 64% were undertaking paid activism. Just over half of the respondents (54.5%) were involved in both paid and unpaid forms of activism. 35.8% were involved only in voluntary/unpaid activism, and 9.7% of respondents were involved only in paid activism (Table 1).

Most respondents were undertaking their activism (paid or unpaid) in user-led charities. The activism described by respondents covered a very broad range, including leadership and governance roles in DPOs, as well as advice, advocacy, policy and campaigning roles. Respondents also described accessibility work in social justice, litigation against discrimination, journalism and creative arts. Others focused on research.

### Dimensionality of the OLBI scale

The OLBI instrument has been developed to measure burnout as a two-dimensional construct comprising Disengagement and Exhaustion, each of them measured by a set of eight balanced items (see Table 2). Although the OLBI has been widely used in the growing research on burnout [51], evidence on the measurement and psychometric properties of the original model is mixed and prior studies have documented difficulties in achieving acceptable model fit while recovering the two proposed axes [see 32, 52, 53 for reviews], and this was also the case for the current study.

Results from a confirmatory factor analysis with two correlated dimensions measured using the original sixteen-item battery suggest that the proposed model does not hold in our sample and fails to achieve acceptable levels of model fit (χ^2^(103) = 346.6, *p* < 0.001; CFI = 0.716; RMSEA = 0.133). The expectation that the sixteen items, containing pro- and con-trait indicators, would consistently measure Disengagement and Exhaustion is not supported by our data. Past studies have reported similar issues and suggested that the use of reversed items might introduce measurement challenges such as the presence of pro-trait and con-trait factorial artefacts [e.g., 41,52].

A second confirmatory factor analysis with two correlated factors, now retaining only the eight pro-trait items, i.e., stronger agreement indicates higher levels of burnout [52] is better supported by the data and reaches appropriate model fit (χ^2^(19) = 33.6, *p* = 0.02; CFI = 0.968; RMSEA = 0.076). Subsequent analyses focus on the results from the eight-item measurement model. Cronbach’s alpha for the two OLBI dimensions, measured with their respective pro-trait items, are *α*_disengagement_ = 0.75 and *α*_exhaustion_ = 0.83. Summated scores are calculated for each OLBI dimension and rescaled to a 0–100 range for ease of presentation. Latent scores were estimated for the two OLBI dimensions using Item Response Theory’s graded response model for polytomous ordinal items. The correlation between the summated scores and their respective latent scores is larger than 0.93 for both OLBI dimensions. In the subsequent analyses, we use the summated score for each OLBI subdimension comprising the pro-trait items only.

### Levels of burnout in the OLBI subscales

**Fig 1:**
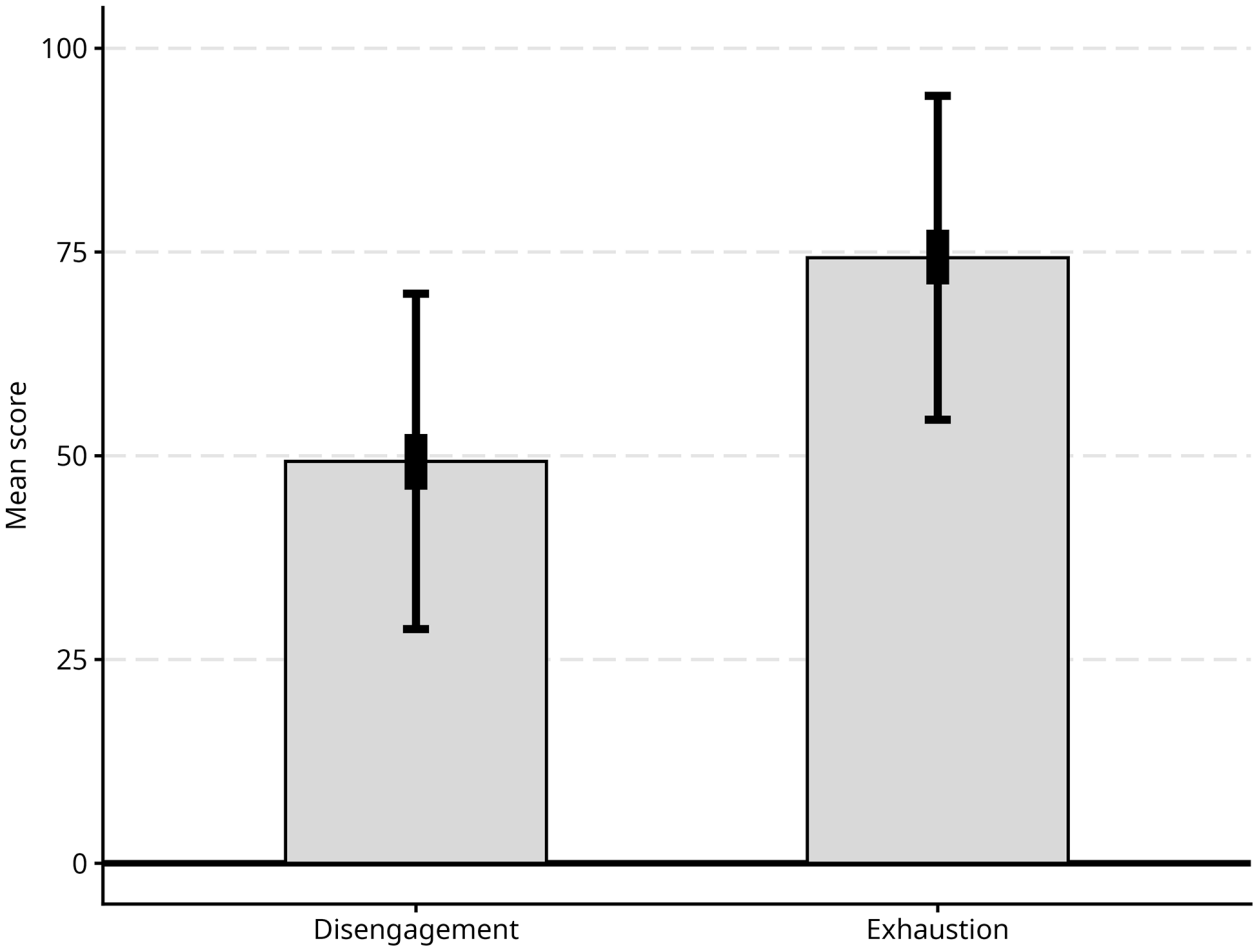
OLBI subscales, mean scores. The thick vertical bars represent the bootstrapped 95% confidence interval for the mean (1,000 resamplings), and the thin vertical bars represent one standard deviation in the observed data.

The two disengagement and exhaustion dimensions are strongly correlated (*ϕ* = 0.83), however, respondents differ significantly in their burnout levels in each of the two OLBI dimensions. Fig 1 reports the mean levels of disengagement and exhaustion scores.

Overall, respondents tend to be significantly more exhausted than disengaged: the mean score for the disengagement subscale is 49.25 and the mean score for exhaustion is 74.31, a 25-point difference (Δ = 25.06, s.e = 2.47, 95% CI = [20.22, 29.91], *p* < 0.001). Likewise, the Wilcoxon rank sum test rejects the null hypothesis of equal location for the two distributions (W = 232, p < 0.001); in other words, the scores of the exhaustion subscale are generally concentrated at higher scores than those on the disengagement subscale.

While the original OLBI instrument does not categorise scores as low, medium or high burnout, it is illustrative that the median score is 50 for the disengagement score and 75 for the exhaustion score on a 0–100 scale, that is, half the respondents score 75 or higher in exhaustion. Recent studies using OLBI have characterised scores above the 75th percentile as relating to high levels of burnout [54].

### Burnout and impairment types

Disability exists and intersects within intersectional identities, which are also made up of other socio-demographic features like gender, class, race and ethnicity, sexuality, religion and more [55]. Because Disability is so often left out, including in key intersectional texts [56,57], the authors have centred disability in the survey design. Therefore, the key relevant identity for sampling is that of disability activists, and the second question on the survey, after asking how they fit the description of disability activist, is to ask which impairments reflect the respondents’ experience of disability. The intention is not to medicalise people’s impairments, but to recognise that a range of impairments [58] and lived experiences may impact on the experience of burnout in different ways.

The data shows that some impairment types are associated with higher levels of burnout in a way that most of the other socio-demographic categories are not, as will be shown below.

Results show that a number of impairments are correlated with higher levels of burnout, primarily in relation to the exhaustion subscale. Respondents reporting four out of the nine impairments covered in the survey – energy-limiting conditions (Δ = 11.6, s.e = 3.22, 95% CI = [5.14, 18.14], *p* < 0.001), long-term health conditions (Δ = 7.2, s.e. = 3.39, 95% CI = [1.10, 13.66], *p* = 0.03), mental health service user/survivor (Δ = 8.8, s.e = 3.37, 95% CI = [2.22, 15.54], *p* = 0.01), physical impairment (Δ = 7.1, s.e. = 3.37, 95% CI [0.13, 13.24], p = 0.04) – have higher levels of exhaustion compared to respondents who did not report those impairments. In contrast, those reporting Deaf/hearing impairment are less burned out than their counterparts not experiencing this impairment (Δ = −11, s.e = 5.15, 95% CI = [−21.08, 1.00], p = 0.02). While it could be tempting to try to draw conclusions about impairment-specific impact on exhaustion from this data, it must be noted that the overall level of exhaustion across the sample is relatively high, with a median of 75 points, which makes those differences relatively modest in the 7−11% range. For disengagement, only those who reported being a mental health service user/survivor (Δ = 7.7, s.e = 3.40, 95% CI = [1.17, 14.34], *p* = 0.04) or who reported neurodiversity (Δ = 6.3, s.e. = 3.54, 95% CI = [−0.42, 13.18], *p* = 0.09) have higher scores in the subdimension vis-a-vis those who do not report experiences with those impairments. This is interesting because, although the two OLBI subdimensions of burnout are strongly correlated, the incidences of disengagement and exhaustion are seemingly affected by different factors [51,59].

**Fig 2:**
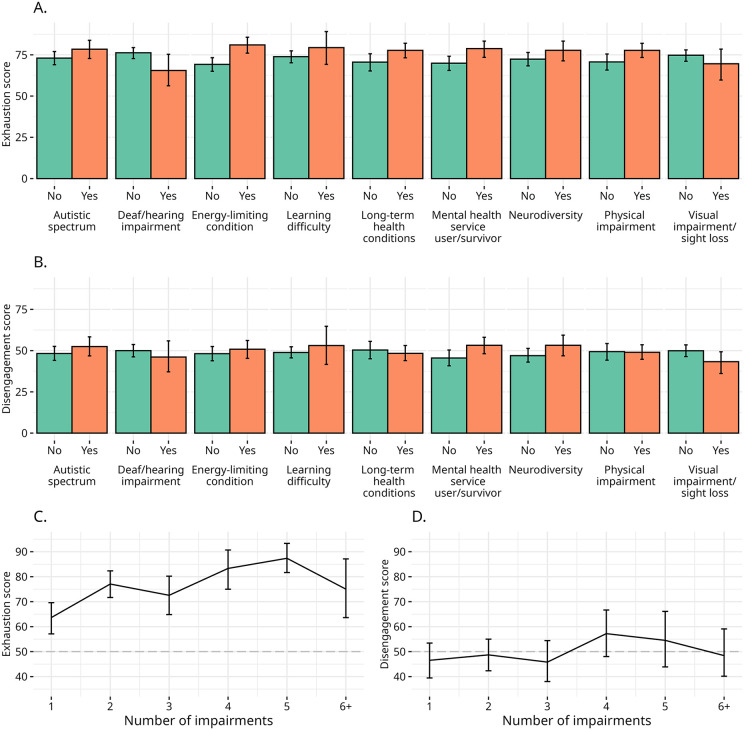
Impairments and burnout. Panels A and B report, respectively, the mean scores for the exhaustion and disengagement subscales for respondents who do and do not declare experiencing the named impairment. Panels C and D report, respectively, the mean scores for the exhaustion and disengagement subscales per the cumulative number of impairments declared by respondents. The number of declared impairments ranges from 1 to 8; because only one respondent declared seven and one respondent declared eight impairments, they are merged with respondents who declared six or more impairments. Vertical bars indicate the 95% bootstrapped confidence interval for the means based on 1,000 resamplings.

The cumulative impact of multiple impairments on burnout is also stronger with regard to exhaustion compared to disengagement. While the correlation between the number of impairments and disengagement is mild at best (Kendall’s *τ* = 0.07, p = 0.3), the correlation is threefold stronger between the number of impairments and exhaustion (Kendall’s *τ* = 0.26, p < 0.001). Visual inspection of Panel B in Fig 2 shows that the mean levels of disengagement remain mostly flat around 50 points across numbers of impairments, whereas mean levels of exhaustion are overall higher and tend to increase with the number of impairments.

We can deduce that levels of exhaustion are correlated with the number of impairments and even with some lived experiences of impairment, however, we cannot also claim that these correlations exist with the experience of burnout overall. In fact, the results push us to resist impairment-specific conceptualisations of burnout, for example of Autistic people, [10,60] and rather to explore what is happening in the gap between the exhaustion and disengagement scores. If we understand burnout as the expression of a political phenomenon [61], rather than as a medical syndrome [39], it becomes clearer how disability activists in the sample can be burnt out but not necessarily scoring highly on the disengagement scale, as many still appear to want to carry on with their activism.

### Socio-demographic features of burnout

Overall, socio-demographics played a relatively minor role in explaining differences in burnout levels. Age is the only socio-demographic variable with consistent associations with OLBI scores, with younger respondents reporting higher levels of both disengagement and exhaustion.

*Age*. The one respondent who answered “prefer not to say” to the age variable is removed from analysis when the age variable is used – i.e., in the bivariate analysis involving age as well as in the regression models. In these two instances, the number of valid cases is 133. Age is the most important sociodemographic variable to explain differences in burnout. Younger respondents report overall higher levels of exhaustion and disengagement compared to the other groups.

The differences between the younger and the older respondents achieve statistical significance at p < 0.05 in five pairwise comparisons and significant at p = 0.10 in one. Noticeably, the difference between respondents aged 18–34 and those aged 35–44 years old is about 7 points (Δ = −7.4, s.e = 4.24, 95% CI = [−16.47, 0.57], *p* = 0.07) for exhaustion and 11 points (Δ = 11.52, s.e = 4.66, 95% CI = [−20.29, 2.11], *p* = 0.02) for disengagement, and the difference between the younger cohort and those aged 55 or older is 16 points for disengagement (Δ = −15.91, s.e = 4.49, 95% CI = [−24.28, −6.55], *p* < 0) and 18 points (Δ = 17.97, s.e = 4.50, 95% CI = [−27.03, −9,28], *p* < 0) for exhaustion. On the other hand, the pairwise differences among 35–44, 45–54, and 55 + years old are overall smaller and non-significant.

This finding aligns with other literature which highlights how younger Disabled People can feel less included within the Disabled People’s Movement, and that there is a lack of accessible and safe spaces for young Disabled People to articulate ideas and visions within the Movement’s activist spaces [62]. A 2023 survey with young Disabled people identified that they want to have a voice within the Disabled People’s Movement, but that they lack time, energy and confidence [63]. Even more recent research with disabled students identified that they typically have higher levels of burnout that non-disabled students [14,64].

*Gender*. The differences between women and men are negligible for both exhaustion (Δ = 0.1, s.e = 3.90, 95% CI = [−7.59–7.93], *p* = 0.98) and disengagement (Δ = −3.6, s.e = 3.79, 95% CI = [−10.89–3.47], *p* = 0.35). We know that Disabled women’s experiences of the world generally and their impairments in particular are different with higher rates of abuse, hardship and caring responsibilities compared to both Disabled men and non-Disabled women [65,66]. However, these real life experiences aren’t translating to differences in the levels of burnout between men and women in our sample of disability activists.

Scores for non-binary respondents are higher than for women or men, and the differences between non-binary and the other two groups are around four times larger than the gap between women and men, with the difference in disengagement between non-binary and women being significant (Δ = 12.6, s.e. = 5.73, 95% CI = [1.52–23.86], p = 0.04). This may be driven by the fact that 11 in 12 respondents in this group also indicated that they were neurodiverse and, as we saw earlier, this group was slightly more disengaged than other groups.

*Sexuality*. Sexuality is weakly associated with burnout. Overall, group differences tend to be relatively small and non-significant. The only noticeable exception is that respondents who selected ‘prefer not say’ for sexuality tend to be less exhausted (at *p* < 0.05) compared to respondents who selected heterosexual, bisexual or other.

*Ethnicity*. In our sample, ethnicity does not stand as a strong predictor of either exhaustion or disengagement. The only pairwise group mean difference that is marginally significant is between white British and other white, where the latter is less exhausted (Δ = −8.5, s.e. = 4.39, 95% CI = [−17.07, – 0.36], p = 0.054).

This finding surprised the authors given that other research has highlighted a significant interaction between race or ethnicity and burnout [20]. In addition, the qualitative element of this study also indicated that there are some additional intersectional challenges faced by disability activists from non-white groups (under review), similarly to how white privilege affects other activist spaces such as feminism [67].

It is possible that the small sample of Black/Global Majority disability activists makes it difficult to identify significant differences in the data so we cannot say for sure whether such differences exist in our sample. Indeed, the small sample size is consistent with other sources that highlight the whiteness of the Movement [68] and of Disability Studies [69] which impacts the involvement and articulation of Black/Global Majority Disabled experiences.

*Religion*: Non-religious respondents have reported higher levels of burnout compared to all other groups. The small number of Muslim respondents and the diffuse content of “others” prevent meaningful comparisons by religion, however we can see that non-religious respondents are more exhausted than Christians (Δ = 14.5, s.e. = 4.78, 95% CI = [5.06–24.29], p = 0.001).

Disability and religion can sometimes be at odds, with Disabled People being variously viewed as linked to curses and witchcraft or to gifts and blessings [70]. There is little research exploring the spiritual or religious beliefs of Disabled People [68]. More broadly, however, research shows that a sense of group belonging achieved through participation in religion or other groups, can be a positive factor in people’s lives [71,72], and perhaps this is mitigating the effects of burnout. On the other hand, research among religious educators emphasises that an inherent disconnect between people’s vocation or calling and the needs of their imperfect workplaces can heighten burnout [73].

**Fig 3:**
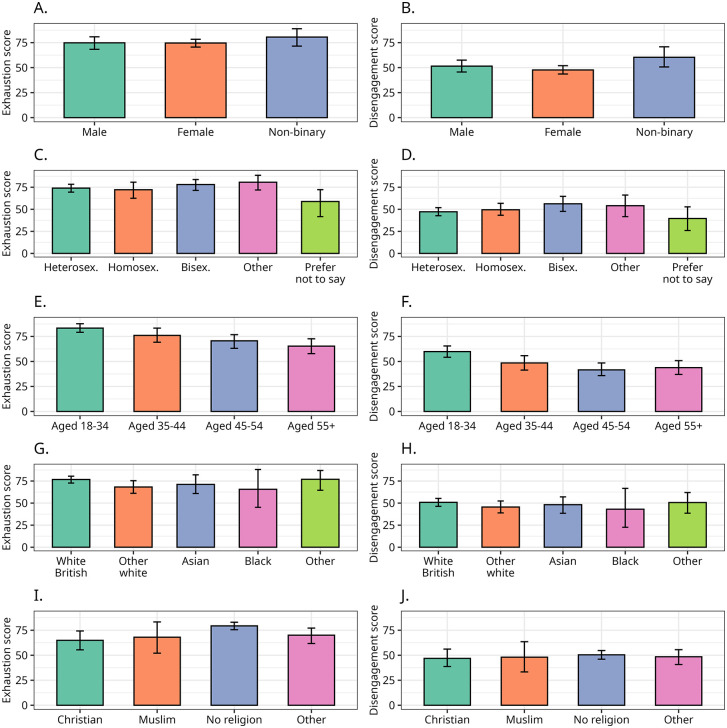
Sociodemographics and burnout. Panels on the left side (A, C, E, G) report the exhaustion subscale mean score for, respectively, gender, age, ethnicity, and religion. Panels on the right side (B, D, F, H) report the disengagement subscale mean score for, respectively, gender, age, ethnicity, and religion. Vertical bars indicate the 95% bootstrapped confidence interval for the means based on 1,000 resamplings.

Mean scores for socio-demographic factors are displayed in Fig 3.

### Perceived social class and burnout

The survey did not include questions such as education and income, but asked participants about their perceived social class. This was because class is no longer easily associated with living standards, income or education [67] and Disabled People are more likely to be disadvantaged in all these areas making traditional class measures less reliable. Therefore, the survey design trusted respondents to self-assess their class.

**Fig 4:**
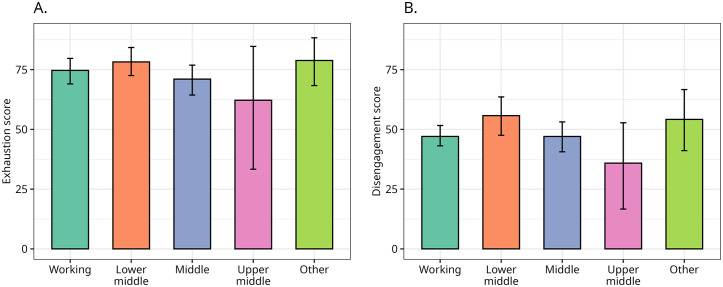
Social class and burnout. Vertical bars indicate the 95% bootstrapped confidence interval for the means based on 1,000 resamplings.

Overall, lower middle class respondents are the most exhausted and disengaged and upper middle class ones are the least. The mean differences among working class, lower middle class and middle class tend not to be significant.The largest difference is found between lower and upper middle classes, with the former being more disengaged by a 20-point margin (Δ = 19.7, s.e. = 10.16, 95% CI = [−40.43–0.31], p = 0.06). Mean scores for social classes are displayed in Fig 4.

These differences are supported by other literature which explores how intersectional differences are understood in the Disabled People’s Movement and identifies class as a key focus of identity alongside impairment, in particular linking class with poverty and economic precarity [74].

### Place of activism and burnout

**Fig 5:**
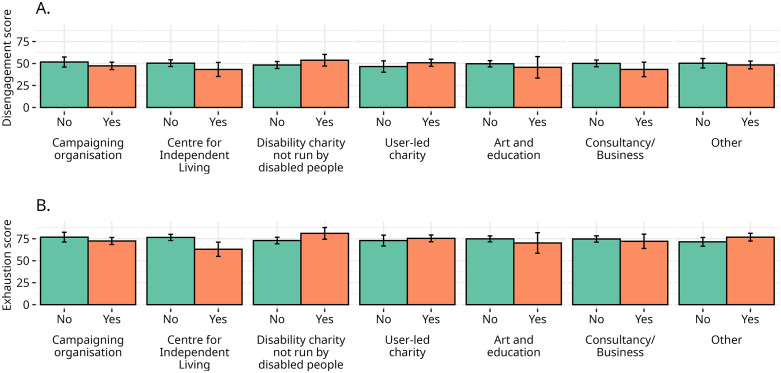
Place of activism and burnout. Paid and unpaid activism of a same type are combined. Vertical bars indicate the 95% bootstrapped confidence interval for the means based on 1,000 resamplings.

Different places of disability activism seem to have diverse impacts on burnout (Fig 5).

Regarding disengagement, respondents who declared involvement in campaigning organisations, centres of independent living, art or education, consultancy or business, reported lower levels of disengagement compared to respondents who did not, whereas those involved in disability charities not run by Disabled people and user-led charities reported somewhat higher levels of disengagement. None of the mean differences for disengagement per type of activism are statistically significant.

Regarding exhaustion, the same overall pattern is observed. Important to notice, however, is that respondents engaged with Centres of Independent Living reported significantly lower levels of exhaustion compared to respondents who are not involved with Centres for Independent Living (Δ = −13.7, s.e. = 4.66, 95% CI = [−22.82 - −4.28], p < 0.01). On the other hand, respondents involved in charities not run by Disabled people reported higher levels of exhaustion compared to those who are not (Δ = 8.1, s.e. = 3.96, 95% CI = [−0.05–15.30], p = 0.078). For other places of activism, differences in exhaustion levels are milder and not statistically significant.

It is difficult to draw concrete parallels from the literature to explain the unique experiences of disability activists. However, we may be seeing a dynamic whereby higher levels of burnout in certain places and spaces such as disability charities not run by Disabled people may be related to inherent disconnect between the experiences of Disabled and non-Disabled people. Similar to the experiences of racial justice activists of colour who can be undermined and harmed by white racial justice activists [75] perhaps differences can be somewhat explained by the setting of Disabled people’s activism. However, the nature of people’s activism where they are involved in numerous groups and organisations in both paid and unpaid capacities makes it difficult to unpick some of these dynamics.

**Fig 6:**
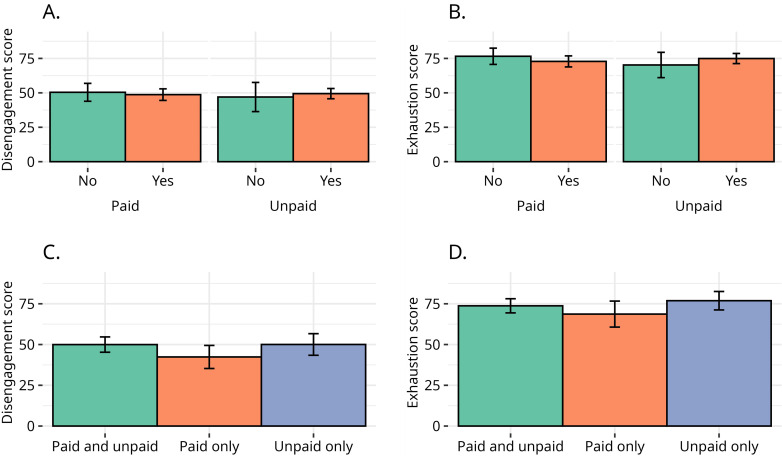
Voluntary vs paid activism and burnout. In Panels A and B, all forms of paid activism are combined as “paid activism”, all forms of unpaid activism are combined as “unpaid activism” and, for each instance, mean levels of disengagement and exhaustion are calculated. “Paid” and “unpaid” are, therefore, not mutually exclusive and the same respondent could be both groups. In Panels C and D, respondents are grouped in mutually exclusive “paid only”, “unpaid only”, and “paid and unpaid” activism. Vertical bars indicate the 95% bootstrapped confidence interval for the means based on 1,000 resamplings.

Involvement in paid or unpaid activism has minor impact on levels of burnout, with involvement in paid activism associated with lower levels of both disengagement and exhaustion, but the differences between paid are small and not significant (Fig 6).

For respondents who could be engaged in both paid and voluntary activism, we further disentangle an eventual double-counting by grouping them in mutually exclusive categories of “paid only”, “unpaid only”, and “paid and unpaid” activism. Some 54.5% of respondents were engaged in both paid and voluntary activism, 35.8% were involved only in voluntary/unpaid activism, and 9.7% of respondents were involved only in paid activism. Respondents engaged in paid activism only are 5–8 points less exhausted and less disengaged compared to both respondents engaged in unpaid activism only as well as those engaged in paid and unpaid activism, which suggests that unpaid activism might be associated with higher levels of burnout. None of the group differences is statistically significant, and other studies suggest that volunteering is a protective factor against burnout [40].

### Multivariate analysis of burnout predictors

The analyses above show the diverse association of a wide range of explanatory variables with burnout, contributing to understanding differences in disengagement and exhaustion levels among the survey respondents; those factors were nevertheless treated separately, despite co-occurring in practice. Using multivariate linear regression models, we assess the effect of impairments, sociodemographics, and place of activism on disengagement (Table 3) and on exhaustion (Table 4).

**Table 3:** Predictors of disengagement.

	Model 1	Model 2	Model 3	Model 4
Intercept	46.18***	63.32***	46.23***	55.18***
	(3.94)	(7.21)	(6.65)	(11.07)
**Impairments**				
Deaf/hearing impairment	−2.23			−1.27
	(4.83)			(5.70)
Physical impairment	0.79			2.36
	(3.90)			(4.28)
Energy-limiting condition	1.81			0.69
	(4.12)			(4.49)
Mental health service user/survivor	6.49			4.03
	(4.05)			(4.93)
Long-term health conditions	−4.40			−8.57+
	(3.91)			(4.57)
Neurodiversity	3.39			3.76
	(4.34)			(5.35)
Autistic spectrum	1.18			−2.84
	(4.62)			(5.50)
Learning difficulty	1.95			−7.64
	(6.23)			(7.48)
Visual impairment/sight loss	−3.51			1.78
	(6.72)			(7.12)
**Age** (reference: 18–34 y.o.)				
35-44 y.o.		−13.84**		−13.88*
		(5.08)		(5.90)
45-54 y.o.		−17.11**		−16.10*
		(5.28)		(6.16)
55 + y.o.		−18.19***		−14.36*
		(5.17)		(6.80)
**Gender** (reference: male)				
Female		−7.66+		−6.98
		(4.14)		(4.56)
Non-binary		1.38		7.11
		(8.45)		(9.37)
Other / prefer not to say		−31.91**		−35.44**
		(11.54)		(13.26)
**Sexuality** (reference: heterosexual)				
Homosexual		1.36		1.76
		(5.56)		(6.03)
Bisexual		4.91		4.90
		(5.18)		(5.70)
Other		−0.42		−2.39
		(6.72)		(7.86)
Prefer not to say		−0.55		1.33
		(7.88)		(8.82)
**Religion** (reference: Christian)				
Muslim		−2.92		2.10
		(9.90)		(11.22)
No religion		0.57		2.45
		(4.97)		(5.51)
Other		3.05		4.71
		(6.05)		(6.74)
**Ethnicity** (reference: British White)				
Other white		−7.49		−6.05
		(4.93)		(5.51)
Asian		−6.11		−9.32
		(7.37)		(8.17)
Black		−11.45		−12.16
		(8.03)		(9.34)
Other		−4.18		−6.84
		(5.90)		(7.02)
**Social class** (reference: working class)				
Lower middle class		7.40		8.78
		(4.95)		(5.93)
Middle class		3.55		4.44
		(4.26)		(4.68)
Upper middle class		−5.95		−5.71
		(8.82)		(10.02)
Other		13.15*		18.73**
		(5.99)		(6.97)
**Place of activism**				
Campaigning Organisation			−5.69	−4.34
			(3.74)	(3.89)
Centre for Independent Living			−7.74	−3.36
			(5.11)	(6.35)
User-led charity			3.15	−1.67
			(4.02)	(4.50)
Disability Charity not run by Disabled People			5.54	0.88
			(5.25)	(5.81)
Education / arts			−7.81	2.22
			(6.52)	(7.40)
Consultancy / Business			−5.50	−5.65
			(5.60)	(6.88)
Other			−3.06	−3.92
			(4.34)	(4.97)
**Paid vs unpaid activism (reference: Paid only)**				
Unpaid activism only			7.58	14.12+
			(6.92)	(7.37)
Paid and unpaid activism			9.85	11.79
			(7.03)	(7.54)
R^2^	0.060	0.292	0.086	0.378
R^2^ Adjusted	0.000	0.158	0.019	0.117
Number of observations	133	133	133	133

+ p < 0.1, * p < 0.05, ** p < 0.01, *** p < 0.001

**Table 4:** Predictors of exhaustion.

	Model 1	Model 2	Model 3	Model 4
Intercept	65.02***	82.62***	72.09***	68.74***
	(3.57)	(6.71)	(6.17)	(9.78)
**Impairments**				
Deaf/hearing impairment	−8.33+			−3.88
	(4.38)			(5.04)
Physical impairment	5.32			4.68
	(3.53)			(3.79)
Energy-limiting condition	7.59*			5.90
	(3.73)			(3.97)
Mental health service user/survivor	4.92			1.81
	(3.67)			(4.36)
Long-term health conditions	2.50			5.62
	(3.54)			(4.04)
Neurodiversity	1.35			−1.87
	(3.93)			(4.73)
Autistic spectrum	2.29			0.33
	(4.19)			(4.86)
Learning difficulty	3.32			−0.04
	(5.65)			(6.61)
Visual impairment/sight loss	−1.66			−4.40
	(6.09)			(6.30)
**Age** (reference: 18–34 y.o.)				
35-44 y.o.		−8.97+		−10.59*
		(4.73)		(5.22)
45-54 y.o.		−11.18*		−7.67
		(4.92)		(5.45)
55 + y.o.		−19.77***		−15.75*
		(4.82)		(6.01)
**Gender** (reference: male)				
Female		−4.52		−3.61
		(3.86)		(4.03)
Non-binary		−6.88		−4.92
		(7.87)		(8.28)
Other/ prefer not to say		−29.81**		−19.76+
		(10.75)		(11.73)
**Sexuality** (reference: heterosexual)				
Homosexual		−3.76		−3.34
		(5.18)		(5.33)
Bisexual		−1.70		−0.45
		(4.83)		(5.04)
Other		0.96		−2.90
		(6.25)		(6.95)
Prefer not to say		−8.00		−9.82
		(7.34)		(7.80)
**Religion** (reference: Christian)				
Muslim		−0.38		2.71
		(9.22)		(9.92)
No religion		12.53**		10.79*
		(4.63)		(4.87)
Other		5.48		5.09
		(5.63)		(5.96)
**Ethnicity** (reference: British White)				
Other white		−9.00+		−9.14+
		(4.59)		(4.87)
Asian		−4.10		−3.40
		(6.87)		(7.22)
Black		−11.22		−7.25
		(7.47)		(8.26)
Other		0.46		1.70
		(5.50)		(6.21)
**Social class** (reference: working class)				
Lower middle class		2.22		5.78
		(4.61)		(5.24)
Middle class		−0.83		2.99
		(3.97)		(4.14)
Upper middle class		−11.48		−16.33+
		(8.22)		(8.86)
Other		5.98		4.20
		(5.58)		(6.16)
**Place of activism**				
Campaigning Organisation			−4.13	−1.87
			(3.47)	(3.44)
Centre for Independent Living			−12.27*	−5.37
			(4.74)	(5.62)
User-led charity			4.14	2.13
			(3.73)	(3.97)
Disability Charity not run by Disabled People			9.28+	7.25
			(4.87)	(5.14)
Education / arts			−13.00*	−7.77
			(6.06)	(6.54)
Consultancy / Business			−4.20	−3.47
			(5.20)	(6.09)
Other			6.61	4.60
			(4.03)	(4.39)
**Paid vs unpaid activism (reference: Paid only)**				
Unpaid activism only			3.03	2.76
			(6.42)	(6.52)
Paid and unpaid activism			0.19	1.91
			(6.53)	(6.67)
R^2^	0.169	0.339	0.152	0.476
R^2^ Adjusted	0.109	0.214	0.090	0.257
Number of observations	133	133	133	133

+ p < 0.1, * p < 0.05, ** p < 0.01, *** p < 0.001

In the descriptive analysis, we reported that, in many cases, associations between explanatory variables and the OLBI subscales were modest and not statistically significant. Results from the regression models corroborate these findings. As shown in Table 3, for the disengagement subscale, coefficient for all impairment types are associated with high levels of uncertainty relative to their regression coefficients, resulting in non-significant results even at *p* < 0.1 (Model 1). Once all other covariates are included in the model, only long-term health conditions remains significant (*p* < 0.01), with learning difficulty now showing a noticeable effect (*p* = 0.064) (Model 4). Among the sociodemographic variables (Model 2), age seems to be the most relevant predictor, associated with a reduction in disengagement. The younger the respondent, the higher the expected level of disengagement, even once all other predictors are taken into consideration (Model 4). Being female has a minor effect in reducing disengagement as well, although the self-declared “other/ prefer not to say” regarding their gender are considerably less disengaged than the male respondents. Even though engagement in most places of activism have negative coefficients, none of them is statistically significant. Interestingly, once other factors are accounted for, being engaged in unpaid only activism is associated with higher levels of disengagement, providing further evidence that unpaid activism might be associated with more disengagement compared to those involved in paid activism or paid and unpaid activism together.

Most regression coefficients in the regression models explaining exhaustion are non-significant (Table 4). However, there are important exceptions to this trend. Among the impairment types, energy-limiting conditions increase exhaustion even when controlled by other impairments (Model 1), but their effects lose significance once controlled by sociodemographics and activism (Model 4). Activism related to education/art and in Centres for Independent Living are associated with lower levels of exhaustion (Model 3), but they lose their significance once controlled for socio-demographic factors (Model 4).

Certain sociodemographic factors stand as relevant explanations for exhaustion (Model 3). Respondents identified as members of the upper middle class are less exhausted (by some sixteen points, *p* = 0.07) compared to other social classes, and non-British White respondents are some nine points less exhausted (*p* = 0.064) than their British White counterparts. Age is a relevant predictor of exhaustion, with younger respondents being more exhausted. Importantly, respondents who stated “no religion” are about ten points more exhausted than their Christian counterparts, even after controlling for other factors. Being engaged in either paid or unpaid activism has no relevant effect on exhaustion.

### Self-assessment of burnout

**Fig 7:**
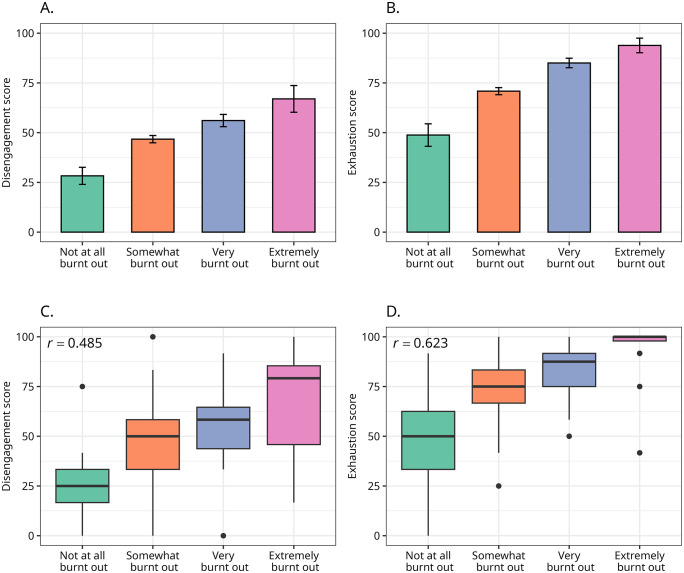
Burnout measured using OLBI by self-assessed burnout. Panels A and B show the average levels of disengagement and exhaustion by the four-point self-assessment of burnout. Vertical bars indicate the 95% bootstrapped confidence interval for the means based on 1,000 resamplings. Panels C and D display boxplots for the distribution of disengagement and exhaustion scores by the four-point self-assessment of burnout; the polyserial correlation for the OLBI subscales and the four-point burnout assessment is shown in the top-left corner of their respective panels.

In addition to the OLBI inventory, the survey also included one question where respondents were asked to self-assess their burnout levels. They were asked “To what extent would you describe yourself as burnt out?” and answered the question using a four-point Likert scale. Eleven per cent of respondents reported “not at all burnt out”, 54.5% reported “somewhat burnt out”, 22.4% declared “very burnt out”, and 12% are self-classified as “extremely burnt out”. Most respondents reported some degree of burnout, with one-third of them reporting to be very or extremely burnt out (Fig 7).

We also found that the self-assessed burnout and the scores in OLBI subscales are correlated, with respondents reporting higher levels of self-assessed burnout also demonstrating higher levels of disengagement and exhaustion. Importantly, the polyserial correlation between self-assessed burnout is more strongly correlated with exhaustion (*ρ* = 0.62) than with disengagement (*ρ* = 0.48).

Combined, these findings suggest that respondents understand *whether* they are burnt out and the *intensity* of the burnout. Moreover, their burnout seems to be more correlated with exhaustion than disengagement.

### Reported drivers of burnout

One hundred and nineteen respondents said that they feel somewhat burnt out, very burnt out or extremely burnt out. These respondents were asked what they think might be making them feel that way through a drop-down list and an open question.

The drop-down list responses were multiple choice across:

- My activism
- My home or family life
- My work outside of my activism
- Other

The combination of responses to these items and their occurrence are reported in Table 5. While some respondents selected a single cause for feeling burnt-out, most commonly their activism (14.3%), 76 respondents (56.7%) selected more than one cause for their burnout, pointing to the compounding nature of factors affecting people. However, their activism remains the most common or co-driver of self-reported burnout across the multiple-choice drop-down list:

**Table 5:** Multiple choice causes of burnout.

Cause of burnout	N	%
My activism (only selection)	21	14.3
My home or family life (only selection)	5	4.2
My work outside of my activism (only selection)	4	3.4
My activism and my home or family life	23	19.3
My activism and my work outside my activism	19	16.0
My home or family life and my work outside my activism	4	3.4
My activism and my home or family life and my work outside my activism	20	16.8
Other causes (in any combination)	32	26.9

“The endless logistics of trying to live as a Disabled person in an inaccessible world and under a hostile government system.” (Survey respondent)

“Exhausted by acting as a mediator between service users and statutory organisations. The work can be emotionally draining as the problems are complex with no easy solutions. There are also things outside of our control. Sometimes when the work feels really hard and then I have challenges outside of work to deal with too, it feels like work and home together can be overwhelming.” (Survey respondent)

This focus by survey respondents on their activism as a driver of burnout makes sense in the context of broader literature on activist burnout whereby activists are seen to often have a sensitivity to injustice, and a heightened sense of personal responsibility to address the injustice [76]. The negative effects of activism are compounded by lived experiences and connections to injustices and oppression [20]. Other driving factors self-identified in open text by respondents who felt burnt-out included 17 who mentioned their health or pain (14.3%). Eleven respondents mentioned the impact of ableism as a driver of feeling burnt-out (9.2%), while 11 also mentioned a lack of resources and accommodation in the workplace, and more broadly in society. A further 11 respondents indicated a general malaise driving their burnout relating to the slow pace of change, constant injustice and the pressures of life generally:

“my life or lack thereof.” (Survey respondent)

We see therefore additional connections with the wider activist burnout literature which posits that physical or emotional health effects may also be linked to burnout [76] and re-emphasises the gruelling impact of being constantly targeted by injustice and oppression [19,20].

A final question asked all 134 respondents to share more about their experience or understanding of burnout. This was a voluntary question which 81 people answered. Key themes which came up as drivers of burnout included external and internal factors including the impact of austerity and government policies, as well as personal exhaustion. These were the two top joint themes, followed by workload and organisational pressures, as well as frustration over the lack of positive change and continued need to keep fighting. This helps to explain that people’s activism per se is not causing burnout, rather that the systems of oppression they face are exhausting [68], and sometimes these systems may be replicated internally within the disability movement.

Broader life challenges such as bereavement, relationship breakdown and financial issues were also identified as drivers of burnout.

Despite the evident challenges experienced by many of the respondents, a small number also mentioned support from and solidarity with others as a protective factor from burnout.

“It’s relentless and exhausting fighting for basic rights. We take such small steps. But I’m energised by the people around me.” (Survey respondent)

The interplay between aggravating and protective factors in disability activist burnout as well as how disability activism is sustained and undermined is further explored in an upcoming paper based on 14 qualitative interviews with disability activists (under review).

## Conclusion

By focussing on disability activists, our study provides a unique contribution to the broader scholarship on burnout. The survey findings raise a number of important points adding to our understanding of burnout among disability activists in the UK. We found that burnout, particularly the dimension of exhaustion, is high.

In exploring the issue of burnout in relation to Disabled People it is helpful to remind ourselves of the routine as well as frequently extreme levels of exclusion, barriers and discrimination they may face in relation to communication, the environment and employment. For disability activists, things like attending a protest, drafting a policy brief or meeting an MP may come with additional barriers because the world is not built to work for them. This experience can also be compounded by overlapping oppressions when Disabled people with intersectional identities are involved in disability activism. This can mean that disability activists not only experience particular difficulties seeking to challenge the issues they face, but routine additional barriers in doing so – personal, social and political. This ‘double or triple whammy’ is particularly pertinent when considering factors related to burnout. As interest in burnout appears to grow, the authors encourage future scholars to continue to consider the experiences of disability activists, as well as the additional barriers that Disabled people face when trying to negotiate even the most routine aspects of civic life. Looking at socio-demographic factors, we found that younger disability activists tend to be more burned out. This finding raises considerable concerns for the future of the Disabled People’s Movement in the UK and further interrogation of this area is something that we suggest for more research.

We also found that the organisations disability activists were engaging with, both paid and unpaid, as part of their activism made some differences to their experiences of burnout. We suggest that further research around where disability activists engage in activism such as with campaign groups, disability charities or disabled-led organisations may be an interesting area to focus on.

A high number of predictors in the regression models are accompanied by large standard errors (i.e., are not statistically significant). Although this might be due to the relatively small sample size, other individual socio-demographic factors may not be the major drivers of burnout. As identified in the analysis of open-ended questions, structural and political aspects – such as governmental policies and practices and organisational structures and resources – are reported by disability activists as important elements to understand burnout. Overall the respondents clearly understand what burnout is and can self-identify its severity in a manner closely aligned to the OLBI scale.

Although disengagement is correlated with exhaustion as predicted by the OLBI model, we find that there is a significant gap in the respective levels of exhaustion and disengagement and these seem to be affected by different factors. We suggest that disability activists remain engaged despite their exhaustion partly because they recognise that their experience is not an individualised experience but rather a symptom of broader structural oppression [77,78]. Additionally, as Disabled People who live the impact of disability oppression daily, it is in some ways impossible to disconnect from the lived reality of ableism or to distance themselves from the real-world impact government policies have on their own lives, such as the decimation of the benefits system. This understanding helps us resist a deficit-based paradigm of burnout and explore the broader implications for causes and potential solutions to burnout, and lays the groundwork for further exploration with disability activists.
